# A Preconditioning Paradox: Contrasting Effects of Initial Phyllosphere and Early Leaf Decomposer Microfungi on Subsequent Colonization by Leaf Decomposing Non-Unit-Restricted Basidiomycetes

**DOI:** 10.3390/jof8090903

**Published:** 2022-08-25

**Authors:** Silvia Bibbo, D. Jean Lodge

**Affiliations:** 1 Plant Ecology & Evolution, Evolutionary Biology Center, Uppsala University, SE-751 05 Uppsala, Sweden; 2 Department of Plant Pathology, Odum School of Ecology, University of Georgia, Athens, GA 30605, USA

**Keywords:** leaf decomposition, mass loss, preconditioning, microfungi, basidiomycetes, fungal cords unit-resource-restricted, non-unit-resource-restricted, white-rot, cord-forming fungi

## Abstract

Fungal interactions during leaf decomposition can facilitate or inhibit other fungi. This experiment focused on whether preconditioning of leaf litter by microfungi that were confined to one leaf (Unit-Restricted) made leaf litter less likely to be colonized and decomposed by basidiomycetes that bind litter into mats (Non-Unit-Restricted) than non-preconditioned litter. Leaves of *Manilkara bidentata* in litterbags were preconditioned by incubating them for 0, 1, 2 or 3 months in flat litter/seed rain baskets 10 cm above the forest floor to avoid colonization by basidiomycete fungi. Preconditioned and non-preconditioned leaves were transferred to 5 replicate basidiomycete fungal mats of *Gymnopus johnstonii* for 6 weeks. Both attachment by basidiomycete fungi and percent mass loss after 6 weeks decreased significantly with increasing preconditioning time. In non-preconditioned leaves, gamma irradiation did not affect mass loss or percent white-rot despite having significantly increased numbers of basidiomycete fungal connections as compared to non-irradiated leaves. In non-preconditioned leaves, more basidiomycetes attachmented to non-irradiated than irradiated leaves suggest facilitation by phyllosphere microfungi. While basidiomycete colonization was initially facilitated by phyllosphere fungi, we inferred that degradation of resource quality led to fewer fungal attachments and less mass loss after 1–3 months of preconditioning by microfungi. The date suggest there is a 1-month time window for basidiomycete fungi to incorporate fallen leaves into their litter mats.

## 1. Introduction

Leaf litter decomposition is a key process that affects nutrient cycling and soil carbon in forest ecosystems with fungi playing the primary role in this process [1]. Many factors influence leaf decomposition including the physical-chemical environment (e.g., temperature and precipitation), litter quality, and the composition of the decomposer community [2,3,4,5]. In wet tropical forests where the physical-chemical environment is relatively constant throughout the year, the decomposer community assumes great relevance in decomposition processes. Leaf decomposer fungi colonize litter either directly from decomposing leaves via root-like structures (hyphal strands, cords, and rhizomorphs), or from a distance via sexual or asexual spores that are airborne or are spread by rainsplash. In humid tropical forests, fungal decomposer species originating from spores produce colonies that are mostly confined to part of a single leaf or twig and are referred to as Unit-Restricted (UR) [6]. Decomposer fungi that colonize leaf litter directly via root-like structures are termed Non-Unit-Restricted (NUR) [6]. As reviewed by Voříšková and Baldrian [7], litter decomposition involves a succession of fungal guilds that utilize different combinations of substrates in their resource base and alter their substratum. Early UR leaf decomposers are primarily Ascomycetes [1,7], though soft-rot species in the Mucorales and Peronosporales may also be present in wet forests [8,9,10]. Early leaf decomposers include phyllosphere fungi (endophytes and latent pathogens) as well as decomposers not found in live leaves. Santana et al. [8] cultured vegetatively dominant fungi from Manilkara bidentata leaves that were decomposed under their source trees for 6 weeks in Puerto Rico and found that half were endophytic fungi and latent pathogenic fungi of the phyllosphere while the others were decomposer fungi that colonized from the forest floor. Direct colonization of leaf litter by NUR basidiomycete fungi in humid tropical forests can occur early or late in decomposition depending on nutrient to carbon ratios [1,11,12,13,14,15,16]. Basidiomycete decomposers can degrade lignin-like compounds and lignocellulose producing a white-rot, which accelerates decomposition [12,15,17]. Dominance of basidiomycete decomposers in middle or late decomposition stages in temperate and boreal forests is associated with increasing accumulation of recalcitrant substrates such as lignin following degradation of more labile components of litter by microfungi, especially Ascomycota [2,7]. Cord-forming basidiomycetes may colonize leaf litter earlier in wet tropical forests than in forests of higher latitude in part because they typically have sclerophyllous leaves that are generally high in recalcitrant compounds [11,12]. In addition, freshly fallen leaves in wet tropical forests often have low P concentrations that resemble those in wood, so cord-forming basidiomycete fungi that can import P accumulated from previously decomposed litter to build biomass in P-deficient litter have a colonization advantage over UR fungi colonizing via spores [11,12]. Santana et al. [8] found in wet tropical forest that a white-rot basidiomycete that survived two rounds of gamma-irradiation in leaf litter in half of the microcosms containing leaves inoculated with early decomposer microfungi lost 21% more mass than leaves in microcosms containing only microfungi. Gamma irradiation was found to eliminate microbes in leaf litter while causing minimal changes to the substrates [8,18]. A few fungi in the Ascomycota also produce white-rot in subtropical forest but they degrade a subset of lignin-like compounds and are less efficient than Basidiomycota in causing mass loss in leaf litter [5].

Fungal priority effects on subsequent colonization and mass loss have been observed during early stages of wood decomposition [19]. Similarly, phyllopsphere fungi may alter substrate use by succeeding fungal decomposers [10,20]. While there are numerous studies of fungal succession during leaf decomposition, we are only aware of one, conducted in temperate forest, that specifically tested the effects of prior fungal colonization of leaf litter by individual early decomposer fungi belonging to the Ascomycetes on subsequent colonization by basidiomycete fungi [10]. They found that *Mycena* sp. caused greater mass loss in beech leaves that were preconditioned by *Xylaria* sp., as compared to either non-preconditioned leaves or ones preconditioned by *Ascochyta* sp. Osono [10] further found that preconditioning of leaves by a fungus in the Ascomycota shifted the substrate utilization by two *Mycena* species in the Basidiomycota from simultaneous removal of lignin and carbohydrates to selective delignification. A few previous studies removed phyllosphere fungi from senesced leaves and compared their decomposition to comparable unsterilized leaves [18,21,22].

In subtropical lower montane forest of Puerto Rico, colonization of freshly fallen leaves by NUR basidiomycete white-rot litter mat formers is typically rapid during the wetter season, beginning within the first 24 h [11,12]. However, leaves not colonized by basidiomycetes within the first month appeared to rarely become incorporated into the basidiomycete litter mats (pers. obs., D.J. Lodge). This unquantified observation, together with the temperate forest study by Osono [10], were the motivation for this experiment. We therefore preconditioned leaf litter for different lengths of time (0, 1, 2 and 3 months), before transferring the litter to basidiomycete litter mats formed by *Gymnopus johnstonii* Murrill) A.W. Wilson, Desjardin and E. Horak, and quantified the number of fungal root-like attachments to the littermat, percent white-rot and mass loss after 6 weeks. We propose two possible mechanisms. First, UR fungi that reach the resource during or before preconditioning may change the composition or the structure of the leaf litter, (e.g., labile and recalcitrant carbon, mineral nutrients, physical structure), either decreasing or increasing the attractiveness of the substrate to basidiomycete decomposers. We refer to these as ‘substrate’ effects (Hypothesis 1). Second, early colonizers of the phyllosphere might interfere with the arrival and colonization of mat-forming basidiomycetes such as *Gymnopus*. We refer to this as the ‘interference’ effect (Hypothesis 2). For example, the interaction between ascomycetes and basidiomycete fungi might lead to the production of an inhibitor made by UR fungi that prevents or limits growth by basidiomycetes. A previous study in montane wet tropical forest in Jamaica [21] that excluded phyllosphere fungi in air dried leaves using propylene oxide sterilization found reduced rather than increased decomposition rates of the leaves, indicating a positive facilitation substrate effect rather than a negative interference effect. Santana et al. [8] found that individual early decomposer microfungi growing together with a basidiomycete in microcosms had additive effects on mass loss. In contrast, sterilization of dogwood leaves from temperate Japan using propylene oxide that were decomposed in microcosms found no effects on mass loss [22]. To determine possible positive or negative effects of early decomposer UR microfungi originating from the phyllosphere on subsequent colonization by a NUR basidiomycete fungus in tropical montane wet forest in Puerto Rico, we gamma-irradiated leaf litter before placing it on basidiomycete litter mats and compared fungal attachment rates, mass loss and percent white-rot to non-irradiated leaves.

## 2. Materials and Methods

### 2.1. Study Site and Species

The experiment was conducted in the Tabonuco Forest along the trail leading from the El Verde Research Station in the El Yunque National Forest located in the Luquillo Mountains of northeast Puerto Rico (18′10″ N, 65′30″ W). Tabonuco forest is classified as a lower montane wet forest with ridges dominated by Dacryodes excelsa (Bursuraceae), Manilkara bidentata (Sapotaceae) and Sloanea berteriana (Elaeocarpaceae) [23,24]. Annual rainfall is approximately 3500 mm and air temperature is relatively constant year round with a low temperature of 21 °C to 24 °C [23]. The experiment took place during the wetter season (June–Octomber 2012) in a former shade-coffee plantation that was abandoned in 1940.

### 2.2. Experimental Design

**Figure 1:**
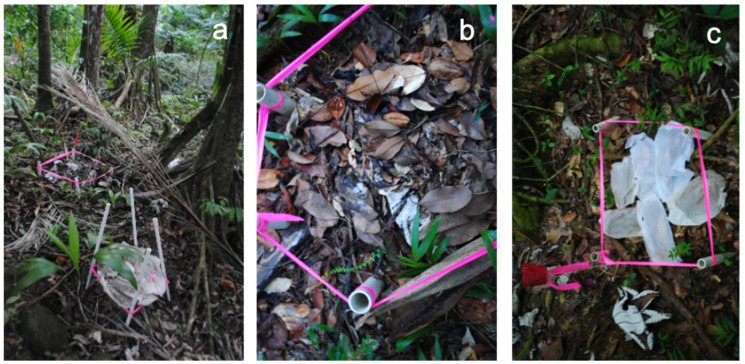
(**a**) Preconditioning of *M. bidentata* leaves suspended in baskets 10 cm above the forest floor. (**b**) Litter mat formed by basidiomycete mycelium, *Gymnopus johnstonii*. (**c**) Leaves in mesh bags placed on *G. johnstonii* litter mat. (Photos by S. Bibbo).

To test the hypothesis that increasing preconditioning time reduces subsequent colonization of litter by basidiomycete fungi (Hypothesis 1), we preconditioned leaves in nets suspended 10 cm above the forest floor (Figure 1a) for 1, 2 and 3 months before placing them directly on 5 white-rot basidiomycete litter mats of G. johnstonii (Figure 1b,c). On 25 May 2012 we simultaneously placed non-preconditioned leaf and irradiated leaf bags directly on white-rot basidiomycete litter mats in a replicated complete block design to reduce variation due to environmental and invertebrate heterogeneity in space and time. One litter bag of each of the 5 treatments was placed on each mat. We selected G. johnstonii because it was the most abundant basidiomycete forming white-rot litter mats at our site [11,13], it can be identified without basidiomes based on the white surficial mycelial fans [13], it causes rapid mass loss [12], and it forms discrete hyphal strands connecting leaves that are easily counted [13]. To test for interference from UR microfungi (Hypothesis 2), we compared gamma irradiated leaves that were not preconditioned or preconditioned for 3 months with comparable non-irradiated leaves. There were 5 treatments with 5 replicates per treatment: no-preconditioning (control), 1-month preconditioning, 2-months preconditioning, 3-month preconditioning, and gamma irradiated leaves that were not preconditioned or preconditioned for 3 months. The treatments were coded as 0 = Control, 1 = 1 month preconditioning, 2 = 2 months preconditioning, 3 = 3 months preconditioning, 4 = gamma-ray control (no preconditioning with gamma-ray), 5 = 3 months preconditioning with gamma-ray) gamma-ray.

### 2.3. Leaf Collection

We collected freshly senescent Manilkara bidentata leaves from the surface of the forest floor immediately after abscission (24 h later maximum), suspended in a net and air dried 9 days in a dehumidified room to constant weight. Oven drying was avoided as this slows decomposition [Lodge, unpublished data; W. Silver, pers. com.]. A subset of air-dried leaves was weighed and oven dried (40 °C) for 48 to estimate the air-dried to oven-dried weight correction factor. Eighty leaf litter bags (25 cm × 15 cm) were constructed of 1-mm nylon mesh. Each bag contained 6 ± 0.5 g of entire, undamaged leaves. The bags were then tagged and weighed again as the pre-treatment mass.

### 2.4. Preconditioning Treatment

Four baskets were built to expose bagged leaf litter to leaching by rain and colonization by unit-restricted microfungi for the three preconditioning treatment times. The baskets were mounted by the trail, under the canopy where Manilkara bidentata trees were dominant. The baskets were suspended about 10 cm from the ground to avoid herbivory and colonization by cord-forming basidiomycete fungi (Figure 1a).

### 2.5. Gamma-Ray Treatment

To test whether sterilizing the leaves to eliminate UR microfungi in the fallen leaves (phyllosphere fungi) affected subsequent NUR basidiomycete colonization, we gamma-irradiated non-preconditioned leaves and leaves preconditioned for 3 months. This removed most of the microorganism present on the leaf litter without changing the leaf composition and structure [18]. We used a custom-built ^137^Cs irradiator at the University of Puerto Rico Medical Sciences Campus, with absorbed doses of 3640 rad and 4090 rad on the first and second exposures, respectively [8].

### 2.6. Measurements

We harvested the litterbags after 6 weeks and counted the number of discrete attachments (i.e., hyphal strands, rhizomorphs and cords) connecting the leaves in the litterbags to the white-rot basidiomycete litter mat below directly in the field. We accomplished this by slowly rolling the litterbag beginning at one edge, counting each attachment as it broke [13]. Lodge et al. [13] found at our site that mass loss in senesced leaves at 14 weeks was significantly predicted by abundance of fungal connections between the senesced litter cohort and forest floor at 7 weeks. Number of attachments were not recorded for non-preconditioned leaves placed on litter mat 1 so we used two different estimates to replace the missing data point: 17 (as in mat 5 with similar % white-rot, 3% vs. 5% compared to 18–34% for the other 3 mats), and 38 (the mean number of fungal attachments to control leaves on mats 2–4). Mass loss (g) during preconditioning was calculated based on initial air-dried weight minus final air-dried weight. Leaves were dried for 9 days at 65C and weighed. The percentage of leaf litter mass loss (%) between treatment and control was calculated as follows: ((initial mass − final mass)/initial mass) × 100 [25]. Percent of leaf area decomposed by white-rot basidiomycetes was estimated after spreading leaves on a 1 cm gridded background, counting the squares more than 50% covered by leaves, then the squares more than 50% covered by bleached leaf surface to obtain cm^2^ total leaf area and white-rot leaf area, respectively [26], then calculating % white-rot as cm^2^ white-rot/cm^2^ total leaf area × 100.

### 2.7. Statistical Analyses

To compare the number of fungal connections between leaves in the litterbags and the underlying basidiomycete NUR fungal litter mat, we first ran an overall ANOVA including the gamma irradiation treatments. This was followed by a 1-way ANOVA to test whether preconditioning exposure (0, 1, 2 and 3 months) to UR microfungi reduced subsequent attachments by NUR basidiomycetes. Percent mass loss violated the assumption of homoscedasticity of variance, so a non-parametric Page test was applied to those data [27]. This was a 1-sided test of equal mass loss across preconditioning times vs. an ordered alternative that mass loss increased with decreasing preconditioning time, with at least one significant inequality.

To explore possible interference by UR microfungi with subsequent colonization by NUR basidiomycetes, we compared non-irradiated leaves of Manilkara bidentata to leaves that were gamma-irradiated prior to placement on white-rot basidiomycete litter mats following 0 or 3 months of preconditioning by unit-restricted (UR) microfungi. These comparisons comprised 5 replicates paired by litter mat and *p* < 0.05. Paired t-tests were used to compare % white-rot and number of fungal attachments to gamma-irradiated vs. non-irradiated leaves preconditioned for the same amount of time (0 and 3 months). Non-parametric Wilcoxon’s Signed Ranks tests were used to compare mass loss in leaves preconditioned for the same amount of time (0 or 3 months and also number of basidiomycete attachments in non-irradiated vs. gamma irradiated leaves [27].

## 3. Results

### 3.1. Mass Loss during Preconditioning

Very little weight loss occurred over the 1–3 months of preconditioning. Mass loss from the initial 6 g was 0.3, 0.6 and 0.7 g at 1, 2 and 3 months, respectively (0.5%, 1% and 1.2%, respectively). Mass loss and % mass loss were close to linear over the 3-month exposure to UR microascomycetes and leaching (R^2^ = 0.96, Pearson’s correlation = 0.98).

### 3.2. Pre-Conditioning Effects on NUR Basidiomycete Attachment, % White-Rot and Mass Loss

**Figure 2:**
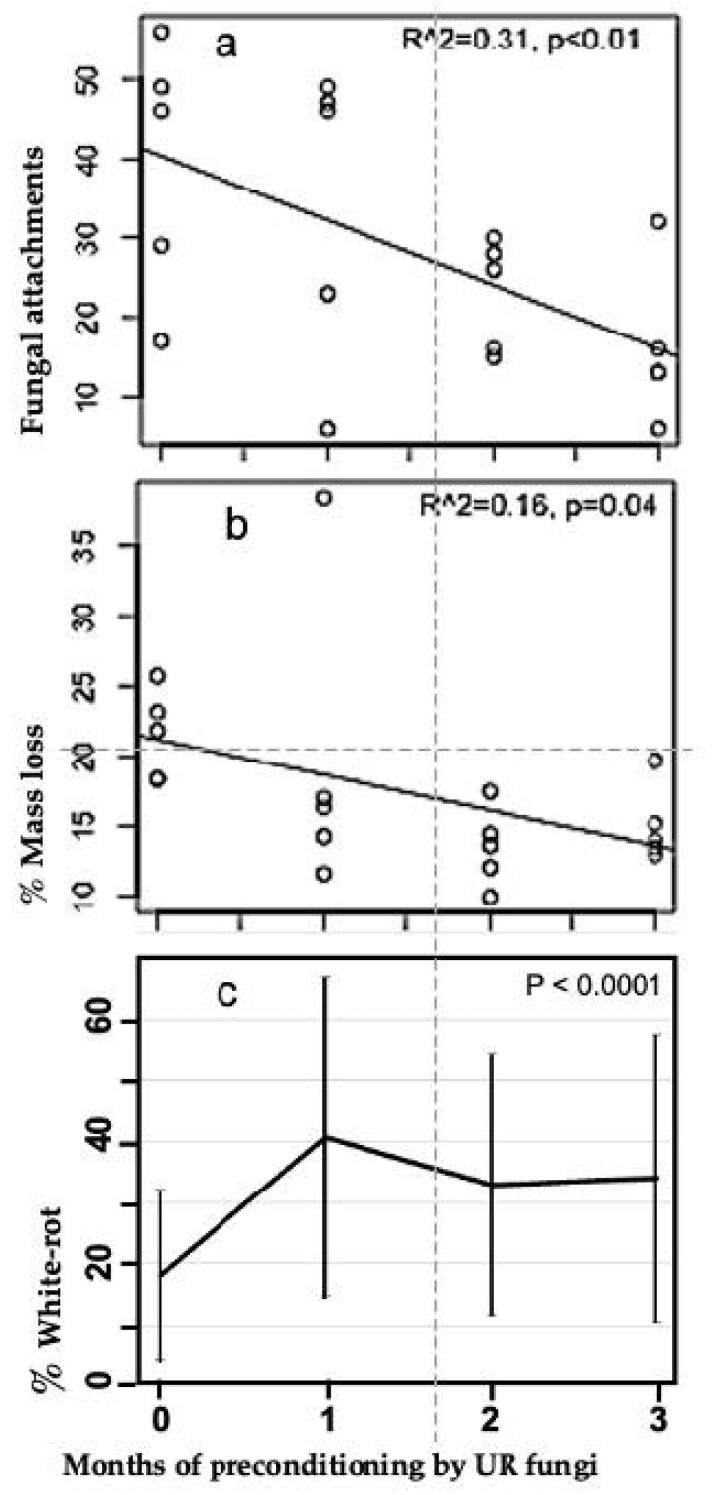
Basidiomycete fungal attachments, mass loss and % white-rot in Manilkara bidentata leaves exposed to 0, 1, 2 and 3 months of pre-conditioning by unit restricted (UR) microfungi and measured 6 weeks after placement on white-rot fungal litter mats in wet tropical forest at El Verde, Puerto Rico. (**a**) Number of basidiomycete fungal attachments (hyphal strands, cords and rhizomorphs) connecting leaves in litterbags to the white-rot litter mat below. (**b**) % mass loss. (**c**) % white-rot (means +/− SD).

Both the numbers of basidiomycete fungal attachments and mass loss in M. bidentata leaf litter decreased with increasing pre-conditioning time (Figure 2a,b). Linear regression showed that about a third of the variation in fungal attachments was related to preconditioning time, and was significant (R^2^ = 0.31, *p* < 0.01). Similarly, preconditioning time was highly significant in the overall ANOVA (Table 1). A 1-way ANOVA for preconditioning time vs. basidiomycete fungal attachment (excluding the gamma irradiation treatment) was also highly significant (*p* < 0.0001). The largest change in basidiomycete fungal attachments occurred between 0 and 1 month of preconditioning (Figure 2a). Similarly, % white-rot after 6 weeks of decomposition on the litter mats vs. pre-conditioning time was highly significant (1-way ANOVA; *p* < 0.0001) and the significant change occurred between 0 and 1 month of preconditioning (Figure 2c). The non-parametric ANOVA (Page Test) used to analyze mass loss vs. preconditioning time was significant (*p* = exactly 0.01) in support of the one-sided hypothesis that mass loss is greater with less preconditioning time.

### 3.3. Basidiomycete Fungal Attachments and Mass Loss in Gamma-Irradiated Leaves vs. Control

**Figure 3:**
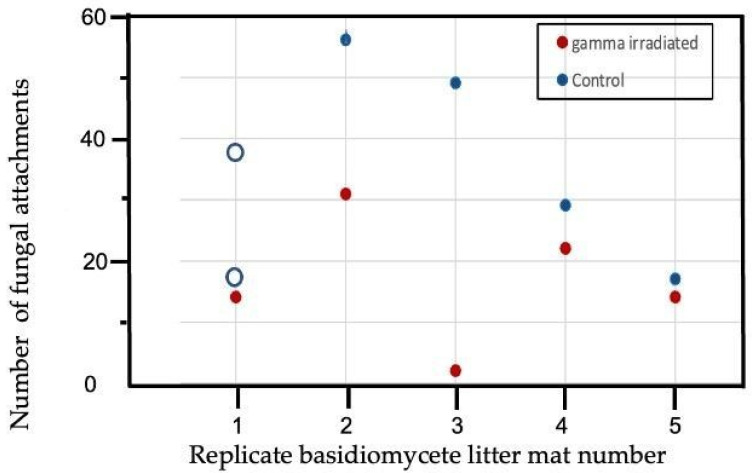
Number of non-unit-restricted basidiomycete attachments to leaves after 6 weeks incubation on Gymnopus johnstonii white-rot leaf litter mats in non-preconditioned leaves of Manilkara bidentata following gamma irradiation versus non-irradiated controls. Number of attachments for the control litter on mat 1 was missing data so a sensitivity analysis was run on the Wilcoxon’s Signed Ranks test using two different estimates of fungal attachments, shown with open circles: 17, the number observed for mat 5 which had similarly low (3–5%) percent white-rot, and 38, which is the mean number of attachments for leaves on the other 4 litter mats.

The senesced air-dried M. bidentata leaves that were not preconditioned (Control) lost a mean of 21.47% +/− 3.17 of their mass in 6 weeks after placement on basidiomycete white-rot litter mats as compared to 19.79% +/− 1.72 mass loss in the leaves that were gamma irradiated prior to placement on the white-rot litter mats. For the leaves preconditioned for 3 months, non-irradiated leaves lost 15.03% +/− 2.71 as compared to 14.75% +/− 2.32 in leaves that were gamma-irradiated after 3 months of preconditioning, but there was one outlier. Wilcoxon’s Signed Ranks tests indicated there were no significant differences in mass loss between irradiated and non-irradiated leaf treatments in either non-preconditioned leaves or leaves preconditioned for 3 months. However, in non-preconditioned leaves, the number of basidiomycete fungal connections was significantly higher in the non-irradiated leaves than in the gamma-irradiated leaves (Figure 3; Wilcoxon’s Signed Ranks test, *p* = 0.031) using both estimates to replace the missing data for the control leaves on one litter mat. The estimates for number of fungal attachments used were 17 (as in mat 5 with similar % white-rot, 3% vs. 5%) and 38 (the mean number of fungal attachments to control leaves on mats 2–4). The number of fungal attachments did not differ between irradiated and non-irradiated leaves that had been preconditioned for 3 months (Supplementary Figure S1). Thus gamma-irradiation only significantly affected (decreased) colonization by basidiomycete fungi of non-preconditioned leaves (i.e., freshly fallen air-dried leaves), while % mass loss was unaffected with either 0 or 3 months of preconditioning.

## 4. Discussion

### 4.1. Interference Effects—Do Unit-Restricted Fungi Inhibit Basidiomycete Colonization?

We negated our second hypothesis that UR fungi interfere with colonization by NUR basidiomycete fungi. Elimination of UR later-stage microfungal decomposers after 3 months of preconditioning had no affect numbers of NUR basidiomycete attachments when placed on white-rot litter mats. Additionally, contrary to H2, elimination of UR early stage phyllosphere microfungi from fresh leaf litter using gamma-irradiation apparently decreased instead of stimulating colonization by NUR basidiomycete fungi. Percent white-rot was also lowest in the absence of preconditioning by microfungi. These observations suggest that preconditioning by UR phyllosphere microfungi during the first month facilitate rather than inhibit white-rot by NUR basidiomycete fungi in wet tropical forest.

### 4.2. Substrate Effects—Preconditioning by UR Affects NUR Fungi through Changes Litter Quality

The facilitation of NUR basidiomycete colonization by UR phyllosphere fungi we observed is consistent with our first hypothesis that colonization by later decomposers is influenced via changes in resource quality mediated by activities of earlier decomposers, though the effect was facilitation of NUR fungi by UR rather than inhibition via resource depletion which we had expected. Using unsterilized leaves, the number of fungal attachments by basidiomycete fungi decreased significantly with increasing preconditioning time, which is consistent with our first hypothesis and prior observations. Correspondingly, percent mass loss also decreased with increasing time of preconditioning by UR microfungi. Little mass loss occurred during preconditioning (<1.2%) only accounting for a tenth of the ca. 10% decrease in mass loss with increasing preconditioning time over 3 months. Together, these data are consistent with our first hypothesis that a decrease in substrate quality during preconditioning by UR fungi as contributing to the decrease in the number of NUR basidiomycete fungal attachments and mass loss.

Changes in fungal community composition during decomposition have previously been found to be associated with changes in resource quality [10,19], which is consistent with our substrate hypothesis. Osono [10] showed in a microcosm experiment that soluble carbohydrates had the largest decrease in chemical constituents during 8 weeks of preconditioning by Xylaria and Ascochyta, but only a slight decrease in percent N and no consistent pattern in percent lignin or total carbohydrate. In a field experiment at our site in tropical lower montane wet forest of Puerto Rico, leaching losses of N, P and K from leaf litter are high during the first 5 weeks of exposure [28], especially in the absence of basidiomycete fungi that recycle nutrients from lower to upper litter layers [13,28]. We hypothesize that leaching of mineral nutrients and decreases in soluble carbohydrates during preconditioning may be responsible for decreasing connectivity by basidiomycete white-rot fungi and decreasing mass loss based on nutrient leaching data from our site [28] and carbohydrate analyses in Japan by Osono [10].

Successional facilitation during longer periods of leaf decomposition has previously been found in tropical montane rain forest by Tanner [21], tropical lowland forest by Hedger [29] and in early or longer-term decomposition of temperate forest leaf litter by Osono [10], Voříšková and Baldrian [7] and Schneider et al. [1]. Osono [10] found in temperate forest of Japan that *Mycena* sp. caused significantly greater mass loss in leaf litter preconditioned by *Xylaria* sp. and *Ascochyta* sp. than control litter that was not preconditioned, but there was either no effect or a negative effect of preconditioning by 11 other species. Santana et al. [8] also found positive, additive mass loss effects of individual early leaf decomposer microfungi together with a basidiomycete, but the UR microfungi originating from the same or related leaf species as the decomposing substratum caused significantly greater mass loss than microfungi that were mismatched by source and substratum.

We know from previous comparisons of endophytic and decomposer fungi of M. bidentata leaves at our site in premontane wet forest in Puerto Rico that except for a few generalist Xylaria species and Phomopsis spp, there is rapid turnover and replacement of the initial phyllosphere mycota in fallen leaves during the first 2–3 weeks of decomposition [8,30,31]. Voříšková and Baldrian [7] and Osono [10] also found rapid replacement of phyllosphere fungi in temperate forest. Despite their short period of dominance, previous studies of phyllosphere fungal effects on decomposition [7,10] and the results of our gamma-irradiation treatment of freshly fallen leaves at our site suggest the ephemeral UR phyllosphere species facilitate rather than interfere with subsequent colonization by NUR basidiomycete fungal decomposers. Ours and previous experiments in microcosms [8,10] and in the field [12,13] show that basidiomycete fungi accelerate mass loss in leaf litter. The reduced number of attachments to irradiated leaves by basidiomycete fungi we observed in wet lower montane forest in Puerto Rico is thus consistent with the reduced rate of mass loss in ethylene oxide-sterilized fresh leaf litter found by Tanner [21] in a wet montane forest in Jamaica. On the other hand, the pattern of mineral nutrient leaching from leaf litter at our site indicates peak losses at 5 weeks, and the losses of mineral nutrients together with decreases in soluble carbohydrates shown by Osono [10] in temperate leaf litter in Japan may explain why basidiomycete colonization of leaf litter at our site declines with increasing time of preconditioning by UR microfungi, and is consistent with our resource hypothesis. Thus, there may be a very short optimal time window of 2–4 weeks after litterfall for NUR basidiomycete colonization of freshly fallen leaves in tropical premontane wet forest at our site.

## 5. Conclusions

We negated our second hypothesis as we found no evidence that unit-restricted UR microfungi interfered with subsequent colonization by non-unit-restricted (NUR) basidiomycete decomposers. Contrary to our expectation, initial UR phyllosphere fungi present in freshly fallen leaf litter facilitated colonization by the dominant NUR white-rot basidiomycete fungus in wet tropical forest of Puerto Rico, consistent with our first hypothesis that alteration of the substrate by early decomposers affects colonization by later decomposer fungi. Longer periods of preconditioning by UR microfungi, however, resulted in fewer NUR basidiomycete decomposer fungal attachments and slower mass loss, which is also consistent with our first, substrate hypothesis. In tropical premontane wet forest such as our site there may be a very short period of 2–4 weeks after litterfall when NUR basidiomycete colonization is facilitated by the activity of the initial phyllosphere fungi but before degradation of resource quality via leaching and utilization of soluble carbohydrates. Future studies would benefit from using high-throughput sequencing to determine the dominant fungi, patterns of enzyme production and the timing of microfungal community turnover during the critical first 4–6 weeks after leaf fall.

## Figures and Tables

**Table 1:** Overall ANOVA comparing effect of 0, 1, 2 and 3 months of preconditioning by leaching and unit-restricted microfungal colonization on subsequent Basidiomycete fungal attachment, with and without sterilization by gamma-irradiation after 0 and 3 months preconditioning.

Factor	D.F.	Sum Sq.	Mean Sq.	F	*p* > F
Treatment	4	5089	1272	8.711	0.00025 ***
Residuals	2, 1	3046	145		

*** denotes highly significant difference with *p* < 0.001.

## Data Availability

Data are available on the Luquillo LTER EDI Data Portal: https://portal.edirepository.org/nis/mapbrowse?scope=knb-lter-luq&identifier=232&revision=2.

## Supplementary Materials

The following supporting information can be downloaded at: https://www.mdpi.com/article/10.3390/jof8090903/s1, Figure S1: Number of Basidiomycete Cord Attachments to Leaves Irradiated vs. Non-Irradiated After 3-Months of Preconditioning by Microfungi.
