# Supplementary material for: A Preconditioning Paradox: Contrasting Effects of Initial Phyllosphere and Early Leaf Decomposer Microfungi on Subsequent Colonization by Leaf Decomposing Non-Unit-Restricted Basidiomycetes
Source: J Fungi (Basel). 2022 Aug 25;8(9):903. doi: 10.3390/jof8090903 (PMC9501227; doi:10.3390/jof8090903)
Supplement: Supplementary file 1 [file jof-08-00903-s001.zip › jof-1866767-supplementary.pdf]

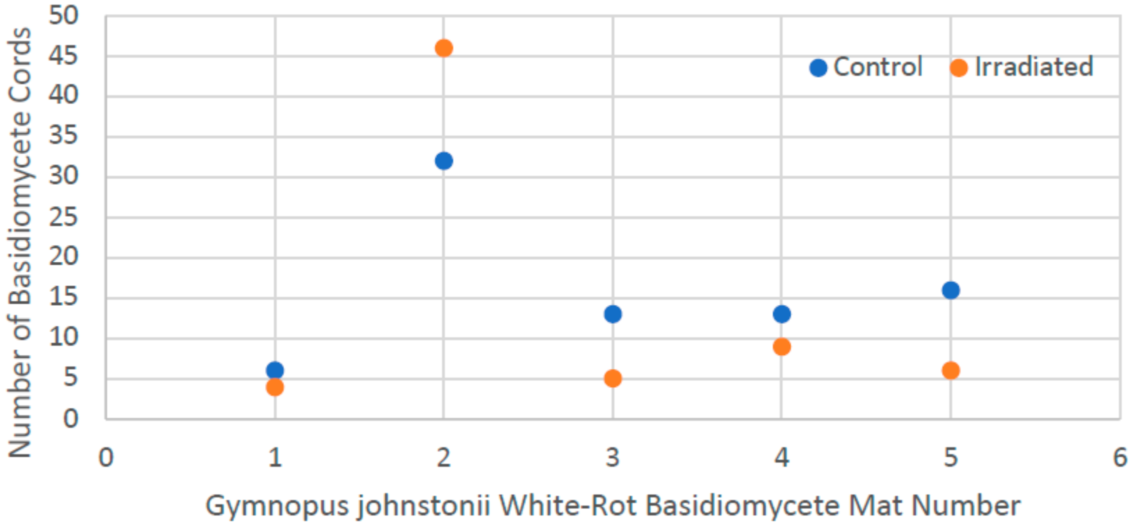

Figure S1. Number of Basidiomycete Cord Attachments to Leaves Irradiated vs. Non-Irradiated After 3-Months of Preconditioning by Microfungi.
